# The College of Nursing and State Registration

**Published:** 1916-04-01

**Authors:** 


### THE COLLEGE OF NURSING AND STATE REGISTRATION.

Matrons and sisters throughout the country have come to take an increasing interest in Mr. Arthur Stanley's proposals to establish a College of Nursing. This movement has now made substantial progress, the position to-day being as follows. Since the issue of Mr. Arthur Stanley's letter in January, conferences have been held between representatives of the State Registrationists, the supporters of the College of Nursing, and representatives of the nurse training schools and societies. The conferences were held on (1) February 23, (2) March 2, and (3) March 24. These conferences were representatively attended, the attendance at the last being the largest. It was then resolved, with two dissentients only, who represented the London Hospital, "that the meeting affirms as the basis of any agreement the necessity of (1) State Registration, (2) a uniform curriculum, and (3) a one-portal examination after such period of training as may be found desirable." As the Chairman said, this decision brought the contestants very much nearer to each other.

On the adoption of the resolution, Professor Glaister, representing the Scottish Association for the Promotion of the State Registration of Nurses, rose and said that his Association would now go in with the College; further, that at a meeting of the Boards of the three principal hospitals in Glasgow, those present were very heartily in favour of the principles of the College, and after what had been stated that day, he and his colleagues would recommend that its scheme be adopted in each of the three great infirmaries of Glasgow. Dr. Comyns Berkeley, representing the Royal British Nurses' Association, which has a Royal Charter, declared that some of them had come there that afternoon to support the College of Nursing, under the firm conviction that that was the quickest way of getting State Registration. Further evidence was forthcoming that other sections of the State Registrationists were in favour of the College of Nursing. The scheme of the College takes power to promote Bills in Parliament in the interests of the nursing profession, their education, organisation, protection, and for their recognition by the State.

The College of Nursing will, therefore, be founded—that is, registered—forthwith, not as a joint-stock company with shares, but as a company limited by guarantee. Of the Council of thirty, Mr. Stanley announced that the following were the names of seventeen who, if passed and appointed, would be the only nominated members. The remaining thirteen would be co-opted, so as to secure that the Council should be thoroughly representative. The names were:—Miss Gill (Edinburgh Infirmary), Miss Haughton (Guy's), Miss Lloyd-Still (St. Thomas's), Miss McIntosh (St. Bartholomew's), Miss Montgomery (Middlesex), Miss Mowat (Whitechapel Infirmary), Miss Paget (Direct Representative C.M.B.), Miss Sparshott (Manchester Royal Infirmary), Miss Swift (Matron-in-Chief, Joint War Committee), Miss Vincent (Leicester Royal Infirmary), Miss Seymour Yapp (Poor-Law Hospital, Ashton-under-Lyne), Dr. Jane Walker, Dr. Comyns Berkeley, M.D., F.R.C.P., Sir Cooper Perry, M.D., F.R.C.P., the Hon. Arthur Stanley, M.P., M.V.O., and Dr. Horace George Turney, M.A., M.D. These names include representatives of important London, Scottish, and provincial training schools for nurses, of the medical profession, State Registrationists, midwives, the Joint War Committee, Poor-Law, and others. When the thirteen vacancies are filled up, Ireland and Wales, and representatives of all important branches of nursing, will no doubt find a place on the Council.

Originally Mr. Stanley drew attention to a strong point of difference amongst those who advocate State Registration. He, like the representatives of the Royal British Nurses' Association and the most important interests in Scotland, with many other authorities of influence throughout the country, holds that the State Registrationists have hitherto made the grave mistake of putting the cart before the horse. Instead of going to Parliament and saying, "Give us power to make a State Register," and then having a series of controversies in Parliament and elsewhere as to what the Register shall be, the supporters of the College of Nursing hold that the desired object would be much more likely to be attained if all these controversies were to be first settled among the different persons concerned, and they were then to go to Parliament and say, "We have formed our Register: give us Parliamentary sanction for that Register." The formation of the College of Nursing will enable the nursing authorities to unite to set up a register, and then, when it is properly formed, to go to Parliament and to ask for Parliamentary sanction. Mr. Stanley holds the opinion that, if this course is taken, recognition will be granted even in war-time. Mr. Stanley, speaking to the whole nursing world, declares: "If we set our house in order first," in the way suggested, "and then go to Parliament, I feel absolutely certain that we shall get what we want."

Another strong point made in favour of the College is that, although it is necessary to start with a Council, some of whom must at the outset be nominated members, yet, as the regulations provide that of that Council two-thirds of the members are to be matrons or nurses in the active practice of their profession, and that every year a third of that Council retires, and the elections to fill those vacant places are to be made only by members of the College—that is to say, by the nurses themselves, by a postal ballot—every nurse will have a direct vote for representatives on the Council. At the end of each of the first three years one-third will retire, their places being filled by the votes of the members of the College. In this way, at the end of three years the Council of the College will consist of persons all of whom will have been elected by the votes of its members.

Matrons, sisters, and nurses will naturally wish to know what will be the procedure of the Council of the College of Nursing at the outset. Mr. Stanley stated that the Council, as soon as it is constituted, will at once proceed to issue invitations to representatives of the training schools and other places throughout the country to a big meeting to be held in London. It is hoped that out of that meeting it will be possible to form a big Consultative Board. That Consultative Board will at once proceed, then, with the formation of small sub-committees which will have to deal with questions such as curricula, examinations, and everything else. Mr. Stanley calculates that that work will take at least two months to carry through, and then all will be referred back to the Council for final approval. The final meeting on the 24th ult., as we have already stated, affirmed the principles upon which the College of Nursing will rest, and the meeting was in general agreement with Mr. Stanley in thinking that the sooner the College of Nursing gets to active work the better it will be for the nursing profession. Our counsel to matrons and hospital committees is to consider at once who will be the best, most knowledgeable, and most competent representative of their training school and hospital to appoint to attend the great meeting in London which Mr. Arthur Stanley will summon to assemble at St. Thomas's Hospital at 3 p.m. on Friday, April 7th inst.

Further, it may be interesting to bear in mind that Mr. Stanley hopes that the College may find it possible to draw a definite line of demarcation between the trained nurse and the woman who is partly trained. Its Council, elected, as it will be, entirely by the votes of the trained nurses as a body representative of all sections, should prove the best possible authority to settle what shall be the examinations and what shall be the certificates not only in their own branch of the profession, but in the lower branches, the untrained women. Another point is that the Council will not train nurses at present. The Council will, however, have power to adopt that course should it be found desirable at any time.

Much time was taken up at the meeting on the 24th ult. by a discussion raised by some of those in favour of State Registration, of whom Major Chapple, M.P., was the chief. Major Chapple moved that a committee be formed to draw up a Bill for presentation to Parliament embodying State Registration and the establishment of a Nursing College. This was seconded by Mrs.

Bedford Fenwick, and supported by Dr. Mcgregor Robertson. The Chairman, Mr. Stanley, at once ruled that this resolution must be postponed until after the formation of the College of Nursing, adding that when the Council was appointed he would be quite willing to receive a resolution to the effect that the Council be asked, as one of its first duties, to proceed with a Bill. The College of Nursing was being formed to do these things, and it would be quite unreasonable, in his opinion, just when the College was on the point of being formed, to appoint a committee which would have nothing to do with it. The issue being thus joined, a debate followed, in which, in addition to those already mentioned, Dr. Comyns Berkeley and Professor Glaister, with others, took part. The two latter speakers supported the Chairman's view, the other three being in favour of the course laid down in the resolution. Ultimately the Chairman ruled Major Chapple's resolution to be out of order, on the grounds (1) that the meeting had assembled, as the notice indicated, to discuss the formation of a College of Nursing, and (2) that one of the objects of establishing that College is ultimately to obtain State Registration.

Altogether it was an interesting and prolonged debate. In the end Miss Cox-Davies intervened to urge that all present were anxious not to separate with divisions unhealed, for all felt strongly that what is done now should be done in unity. She therefore suggested that a joint committee to consider a Bill for the State Registration of Nurses should emanate from the Council of the College. After further discussion, on the suggestion of Major Chapple the Chairman agreed to give his personal pledge that the first work of the Council, when set up, shall be to form a small committee to confer with Major Chapple and his advisers on the subject of a Bill. This pledge was accepted by the meeting, and a vote of thanks to the Chairman, moved by Major Chapple, was carried with applause.

